# Quality of life of individuals with chronic kidney disease on dialysis

**DOI:** 10.1590/2175-8239-JBN-2018-0152

**Published:** 2019-01-24

**Authors:** Nadaby Maria Jesus, Gracielly Ferreira de Souza, Clesnan Mendes-Rodrigues, Omar Pereira de Almeida, Deusdélia Dias Magalhães Rodrigues, Cristiane Martins Cunha

**Affiliations:** 1 Universidade Federal de Uberlândia UberlândiaMinas Gerais Brasil Universidade Federal de Uberlândia, Uberlândia, Minas Gerais, Brasil.

**Keywords:** Quality Of Life, Renal Insufficiency, Chronic, Renal Dialysis

## Abstract

**Introduction:**

Chronic kidney disease (CKD) negatively affects the physical and biopsychosocial aspects of the lives of individuals with the disease, thereby affecting the quality of life (QOL) of patients and their families.

**Objectives:**

This study aimed to measure the QOL of individuals with CKD and compare the QOL scores of patients with CKD to the scores of disease-free individuals to find factors associated with better QOL.

**Method:**

The local Ethics Committee approved this cross-sectional study. The study was carried out at a public clinic and a private hemodialysis clinic. Participants were asked to answer the WHOQOL-BREF and a sociodemographic questionnaire. Statistical tests were used according to the variables of interest and significance was attributed to differences with *p*-values < 0.05.

**Results:**

Nearly two thirds (59%) of the case group members were males and 55% did not have a spouse; 53% were seen at a private clinic and 57% had complications. The variables that more significantly affected QOL were smoking (perception of QOL) (*Bi* = - 0.4061; *p* = 0.032), undergoing hemodialysis (general health status) (*Bi* = - 0.3029; *p* = 0.034), and duration of sessions (*Bi* = 0.117; *p* = 0.039) (environmental domain).

**Conclusion:**

The QOL of patients with CKD was significantly lower when compared to controls in the physical and psychological domains. Several variables affected the perception of QOL and should be considered in clinical assessment.

## Introduction

Increased patient morbimortality and negative impacts on the quality of life (QOL) of patients and their families have turned chronic kidney disease (CKD) into a significant public health issue.^1^^,^^2^

CKD gradually compromises renal function through irreversible kidney injury.^3^ The main causes of CKD include hypertension, diabetes mellitus, and glomerulonephritis.^4^ The disease is divided into five stages. Stage 1 is characterized by kidney injury with mild loss of renal function, yet without direct impact on the glomerular filtration rate (GFR). Individuals in the more advanced stage of the disease suffer from kidney failure and have a GFR below 15 mL/min.^5^ Patients in this stage are offered renal replacement therapy (RRT) in the form of hemodialysis or peritoneal dialysis, and may be referred for renal transplantation.^6^

Hemodialysis is currently the most widespread mode of RRT.^7^ Although it is meant to support patient life, RRT - along with CKD - negatively affects the QOL of patients by introducing changes to their habits and daily living in the form of continuous drug therapy, water intake restrictions, time away from work,^4^ physical and nutritional limitations, impaired social and family life,^8^ and dependence on constant clinical outpatient monitoring.^9^ Patients with CKD on RRT also experience declines in their sex lives, existential conflicts, and spiritual distress, which in turn worsen physical and emotional symptoms.^10^ All such repercussions compromise physical, mental, and emotional wellbeing^11^ and worsen QOL.^10^

QOL is a multidimensional entity that includes repercussions on the physical psychological, social, and environmental dimensions, not only in the absence of disease.^3^ Therefore, valid reliable psychometric instruments are required to assess perceived QOL.^12^ The WHOQOL is a scale developed by the World Health Organization (WHO) to rate individual perceptions of QOL and assess QOL of different groups in different circumstances.^12^

Living with CKD requires adaptation and changes to daily routine and habits, which in turn challenge the perceptions individuals have of themselves, their abilities, and the environment they live in.^13^ On account of the various negative impacts of CKD on the lives of patients, it is relevant and desirable to assess QOL to identify affected areas and provide input to interventions devised to improve living conditions and the health of individuals with CKD.

This study aimed to measure the QOL of individuals with CKD on RRT (hemodialysis), compare whether there are differences in the QOL of patients with CKD on hemodialysis in relation to controls, and assess the possible impact of various social, demographic, and clinical variables on patient QOL.

## Method

### Study design, site, and ethics

This cross-sectional analytical descriptive quantitative comparative study is part of a broader set of themed studies and was approved by the local research ethics committee (CAAE 67009117.0.0000.5152). The study was carried out at a public teaching hospital and at a private hemodialysis clinic providing services to the Brazilian public healthcare system (SUS).

### Participants

The convenience consecutive sample of individuals included in this study was divided into case and control groups. The participants were explained the objectives of the study, and after all clarification was given they were asked to sign an informed consent form.

The case group included individuals aged from 18 to 80 years diagnosed with CKD and on RRT (hemodialysis) for more than six months, monitored regularly and with intact cognitive function as rated by a scale to assess discriminating capacity and psychical and mental orientation of individuals in time and space. Subjects hospitalized within less than 30 days from the start of the study, individuals on RRT for less than six months, and patients on peritoneal dialysis were excluded.

The control group included individuals without chronic diseases aged from 18 to 80 years with intact cognitive function. Individuals hospitalized within less than 30 days from the start of the study and subjects diagnosed with infectious diseases were excluded.

### Procedures

Data collection interviews took place from October 2017 to March 2018. The members of the two groups answered the questionnaires after their psychical and mental fitness was assessed based on the criteria described by Pfeifer.^14^

The interviews were held in a private setting during hemodialysis. The following questionnaires were answered: 1) Sociodemographic characterization questionnaire (sex, age, birth place, marital status, years of schooling, household income, number of individuals living in the household, place of residence, type of work and current employment); 2) Clinical assessment questionnaire (medical diagnosis and etiology of CKD, comorbidities, time on hemodialysis, length of hemodialysis sessions, number of hemodialysis sessions per week, complications, and habits); and 3) Brazilian validated version of the WHOQOL-BREF.^15^

The WHOQOL-BREF includes 26 items divided into four life domains (physical, psychological, social, and environmental) and another two questions on QOL and satisfaction with the current health status.^16^ It is a self-explanatory tool with items referring to the last two weeks of life of the interviewees.^17^

The 26 items of the WHOQOL-BREF are rated on a 5-point Likert scale, in which individual perceptions vary in terms of intensity (*not at all* to *an extreme amount*), ability (*not at all* to *completely*), frequency (*never* to *always*), and subjective evaluation (*very dissatisfied* to *very satisfied*; *very poor* to *very good*).^18^ The scores in each domain add up to totals ranging from 0 to 100 points. Higher scores mean better quality of life.^19^

### Statistics

The normality of the continuous variables was tested with the Kolmogorov-Smirnov test with Lilliefors correction. Qualitative variables were dichotomized and presented in the form of absolute and relative frequencies, while numerical variables were presented in the form of absolute or relative frequencies or as mean values and standard error, minimum and maximum values and median values. Case and control groups were compared with the chi-square test of independence with continuity correction. Continuous unpaired data not following a normal distribution were compared with the Mann-Whitney test. Multiple linear regression analysis was used to assess the domains of the WHOQOL-BREF.

Data sets were analyzed using the IBM Statistical Package for the Social Sciences (SPSS) version 20.0. Significance was attributed to differences with a *p*-value < 0.05.

## Results

A total of 114 patients with CKD were initially considered. Fourteen (11%) were excluded for not being able to answer the questionnaires or not meeting the enrollment criteria. Therefore, 100 individuals were included in the case group.

Five (5%) of the 105 individuals considered for the control group refused to join the study. Therefore, 100 subjects were included in the control group.

The groups were predominantly formed by male individuals (59%) with incomplete middle school education (68%) without a spouse (55%). Most (53%) were seen at a private clinic and 57% claimed they had complications connected with CKD. The case group was older (mean age: 53.59 years, SE 1.47) than the control group (mean age: 47.79 years, SE = 1.52) (*p =* 0.004). The control group had higher levels of education, individual and household income (*p <* 0.05) (Table 1).

**Table 1:** Social and demographic profile of individuals with chronic kidney disease (case group) and controls

Variable	Description	Group n (%)			*p^1^*
		Hemodialysis n=100	Controls n=100		
Sex	Male	59 (59)	46 (46)		0.089
	Female	41 (41)	54 (54)		
Place of residence	City of the study	83 (83)	92 (92)		0.087
	Other cities	17 (17)	8 (8)		
Marital status	No spouse	55 (55)	38 (38)		0.023
	Spouse	45 (45)	62 (62)		
Schooling	Incomplete MS	68 (68)	44 (44)		0.001
	MS/college education	32 (32)	56 (56)		
Smoking	No	86 (86)	93 (93)		0.166
	Yes	14 (14)	7 (7)		
Physical activity	No	71 (71)	63 (63)		0.229
	Yes	29 (29)	37 (37)		
Clinic of origin	Public	47 (47)			
	Private	53 (53)			
Complications	No	43 (43)			
	Yes	57 (57)			
Comorbidities	No	16 (16)			
	Yes	84 (84)			
Variable	Hemodialysis n=100		Controls^#^ n=100		*p* ^2^
	Mean ± SE	Min-Max (Median)	Mean ± SE	Min-Max (Median)	
Age (years)	53.59 ± 1.47	21 - 81 (54)	47.79 ± 1.52	20 - 82 (47)	0.004
Schooling (years)	8.41 ± 0.39	0 - 17 (9.5)	9.92 ± 0.36	2 - 20 (11)	0.003
Individual income (BRL)	1584.56 ± 150.17	800 - 10000 (980)	1842.88 ± 151.9	0 - 8000 (1300)	0.018
Household income (BRL)^#^	2712.52 ± 220.78	900 - 11000 (1980)	3441.6 ± 272.2	900 - 22000 (2800)	0.001
Minimum wages	2.52 ± 0.13	1 - 6 (2)	2.96 ± 0.12	1 - 6 (3)	0.004
Persons in the household (number)	2.6 ± 0.15	0 - 7 (2)	2.82 ± 0.15	0 - 6 (3)	0.187
Session length (minutes)	220.25 ± 2.29	150 - 270 (225)			
Time on hemodialysis (months)	5.05 ± 0.43	1 - 19 (4)			
Time since diagnosis (months)	7.16 ± 0.68	1 - 40 (5)			
Comorbidities (number)	1.57 ± 0.1	0 - 4 (1.5)			

Key: SE: standard error; Min: minimum; Max: maximum; MS: middle school; *p*^1^: test-based probability the chi-square continuity correction; *p*^2^: test-based probability the Mann-Whitney;

# n=100 in each group, except for income (n=99 in control group).

Only two domains in the WHOQOL-BREF - physical and psychological - were statistically different when the two groups were compared (*p* < 0.001) (Table 2).

**Table 2:** Comparison between the scores in the domains of the WHOQOL-BREF of individuals with chronic kidney disease (on hemodialysis) and controls

Domain	Hemodialysis n = 100		Controls n = 100		*p* ^1^
	Mean ± SE	Min-Max (Median)	Mean ± SE	Min-Max (Median)	
RQ1	3.88 ± 0.09	1 - 5 (4)	3.99 ± 0.07	1 - 5 (4)	0.697
RQ2	3.47 ± 0.10	1 - 5 (4)	3.68 ± 0.1	1 - 5 (4)	0.114
PD	56.64 ± 1.74	14.29 - 96.43 (57.14)	71.36 ± 1.48	42.86 - 100 (71.43)	< 0.001
PsD	67.5 ± 1.70	25 - 91.67 (70.83)	73.08 ± 1.48	20.83 - 100 (75)	0.034
SD	70.67 ± 1.86	25 - 100 (75)	72.42 ± 1.75	16.67 - 100 (75)	0.644
ED	62.44 ± 1.42	31.25 - 100 (62.5)	60.88 ± 1.35	28.13 - 90.63 (62.5)	0.576

Legend: RQ1: Perception of quality of life; RQ2: Satisfaction with health; DF: Physical domain; DP domain Psychological; DRS social relations domain; DMA domain Environment. Min: minimum; max: maximum; EP: standard error; p: probability.

1 probability based on the Mann Whitney test.

The multiple linear regression model identified to what extent the variables might be potential predictors for better or worse QOL as rated in the WHOQOL-BREF. Table 3 shows that smoking negatively affected the perception of QOL (*Bi* = -0.4061; *p* = 0.032) of both groups.

**Table 3:** Linear regression analysis results for the domains in the WHOQOL-BREF for patients with chronic kidney disease (on hemodialysis) and controls

Domain	Variable 1	*Bi*	SE	*t*	*p*
RQ1	Constant	3.9775	0.061	65.06	< 0.001
	Smoking	-0.4061	0.188	-2.16	0.032
RQ2	Constant	3.3039	0.264	12.5	< 0.001
	Hemodialysis group	-0.3029	0.142	-2.13	0.034
	Sex	-0.2711	0.141	-1.92	0.056
	Age (years)	0.0108	0.005	2.31	0.022
PD	Constant	74.8771	3.674	20.38	< 0.001
	Hemodialysis group	-14.08	2.331	-6.04	< 0.001
	Place of residence	-7.1456	3.441	-2.08	0.039
	Schooling	5.5504	2.346	2.37	0.019
PsD	Constant	76.6234	3.663	20.92	< 0.001
	Group	-5.2591	2.325	-2.26	0.025
	Place of residence	-6.1972	3.431	-1.81	0.072
	Schooling	3.998	2.339	1.71	0.089
SD	Constant	68.8406	1.862	36.98	< 0.001
	Marital status	5.2248	2.539	2.06	0.041
ED	Constant	59.0244	4.618	12.78	< 0.001
	Place of residence	-9.2293	2.822	-3.27	0.001
	Schooling	5.4504	2.131	2.56	0.011
	Age (years)	0.1117	0.065	1.72	0.087
	Individual income (BRL)	0.0016	0.001	2.39	0.018

Key: *Bi*: i-esimal estimation of model parameters; *t*: estimation from Student's t-test; SE: standard error; *p*: probability; RQ1: perception of quality of life; RQ2: satisfaction with health status; PD: physical domain; PsD: psychological domain; SD: social domain; ED: environmental domain.

^1^ Variable codes: Group: (1: patients on hemodialysis, 0: controls); Place of residence (1: city where the study was carried out, 0: other cities); Marital status (1: with spouse, 0: without spouse); Schooling (1: complete middle school or higher, 0: Incomplete middle school or lower); Sex (1: female, 0: male).

Satisfaction with health status was negatively affected in individuals on hemodialysis (*Bi* = - 0.3029; *p* = 0.034). The variables that more adversely affected the physical domain were hemodialysis (*Bi=* -14.07; *p* = < 0.001) and residing in the city where the study was carried out (*Bi* = -7.14; *p* = 0.039). More years of schooling positively affected this domain (*Bi* = 5.55; *p =* 0,.019).

The only factor to significantly - and adversely - affect the psychological and social domains was hemodialysis (*Bi =* -5.26; *p =* 0.025). Only marital status - having a spouse - positively affected this domain (*Bi* = 5.22; *p =* 0.041).

In the two groups, the most significant factor in the environmental domain was more years of schooling (*Bi* = 5.4504; *p =* 0.011) and higher levels of individual income (*Bi* = 0.0016; *p* = 0.018).

Table 4 shows the results of the multiple linear regression analysis of the variables that more significantly impacted the QOL of individuals on hemodialysis. Perception of QOL of individuals on hemodialysis was positively affected by variables marital status - having a spouse - (*Bi* = 0.379; *p* = 0.042) and higher levels of individual income (*Bi* = -0,00018; *p* = 0.006). Undergoing hemodialysis at a public clinic (*Bi* = -0.513; *p* = 0.007) and smoking (*Bi* = -0.527; *p* = 0.039) adversely impacted QOL.

**Table 4:** Linear regression analysis results for the domains in the WHOQOL-BREF of patients with chronic kidney disease on hemodialysis

Domain	Variable 1	*Bi*	SE	*t*	*p*
RQ1	Constant	4.561	0.299	15.24	< 0.001
	Place of residence	-0.454	0.248	-1.83	0.071
	Marital status	0.379	0.184	2.06	0.042
	Schooling	0.375	0.207	1.81	0.073
	Smoking	-0.527	0.252	-2.09	0.039
	Individual income (BRL)	-0,00018	0,000063	-2.79	0.006
	Clinic of origin	-0.513	0.185	-2.78	0.007
RQ2	Constant	4.176	0.281	14.85	< 0.001
	Schooling (years)	-0.049	0.026	-1.86	0.065
	Clinic of origin	-0.626	0.205	-3.05	0.003
PD	Constant	8.318	18.882	0.44	0.661
	Place of residence	-10.461	4.416	-2.37	0.02
	Schooling	10.391	3.931	2.64	0.01
	Age (years)	0.23	0.116	1.98	0.051
	Family income (BRL)	-0.001	0.001	-1.82	0.072
	Number of persons in the household	2.292	1.076	2.13	0.036
	Clinic of origin	-7.024	3.435	-2.05	0.044
	Number of comorbidities	-3.951	1.655	-2.39	0.019
	Session length (minutes)	0.222	0.071	3.15	0.002
PsD	Constant	87.469	5.819	15.03	< 0.001
	Place of residence	-12.989	4.395	-2.96	0.004
	Marital status	5.605	3.19	1.76	0.082
	Schooling	12.368	3.703	3.34	0.001
	Minimum wages	-4.166	1.304	-3.20	0.002
	Clinic of origin	-11.000	3.237	-3.40	0.001
SD	Constant	-3.979	18.904	-0.21	0.834
	Age (years)	0.286	0.121	2.36	0.02
	Number of comorbidities	-3.017	1.807	-1.67	0.098
	Session length (minutes)	0.291	0.077	3.78	< 0.001
ED	Constant	23.95	14.308	1.67	0.097
	Place of residence	-8.243	3.442	-2.39	0.019
	Schooling	11.302	2.846	3.97	< 0.001
	Age (years)	0.367	0.092	3.98	< 0.001
	Number of comorbidities	-2.398	1.327	-1.81	0.074
	Session length (minutes)	0.117	0.056	2.10	0.039

Key: *Bi*: i-esimal estimation of model parameters; *t*: estimation from Student's t-test; SE: standard error; *p*: probability. RQ1: perception of quality of life; RQ2: satisfaction with health status; PD: physical domain; PsD: psychological domain; SD: social domain; ED: environmental domain.

^1^ Variable codes: Group: (1: patients on hemodialysis, 0: controls); Place of residence (1: city where the study was carried out, 0: other cities); Marital status (1: with spouse, 0: without spouse); Schooling (1: complete middle school or higher, 0: Incomplete middle school or lower); Sex (1: female, 0: male).

In the area of satisfaction with health status, undergoing hemodialysis at a public clinic negatively affected QOL (*Bi* = -0.626; *p* = 0.003). The variables adversely affecting the physical domain were greater number of comorbidities (*Bi* = -3.951; *p* = 0.019), undergoing hemodialysis at a public clinic (*Bi* = -7.024; *p* = 0.044), and residing in the city where the study was carried out (*Bi* = -10.461; *p* = 0.02). However, higher level of education (*Bi* = 10.391; *p* = 0.01), older age (*Bi* = 0.23; *p* = 0.05), greater number of persons in the household (*Bi* = 2.292; *p* = 0.036), and longer duration of hemodialysis sessions (*Bi* = 0,222; *p* = 0,002) positively affected this domain.

The only variable to positively affect the psychological domain was having a higher level of education (*Bi* = 12.368; *p* = 0.001). Residing in the city where the study was carried out (*Bi* = -12.989; *p* = 0.004), higher income (*Bi* = -4.166; *p* = 0.002), and undergoing hemodialysis at a public clinic (*Bi* = -11.0; *p =* 0.001) negatively affected QOL in this domain.

Age (*Bi* = 0.286; *p =* 0.02) and longer hemodialysis sessions (*Bi* = 0.291; *p =* < 0.001) positively affected social relations. Higher levels of education (*Bi* = 11.302; *p =* 0.001), older age (*Bi* = 0.367; *p =* < 0.001), and longer hemodialysis sesisons (*Bi* = 0.117; *p =* 0.039) positively affected the environmental domain. However, residing in the same city of the hemodialysis center (*Bi* = -8.243; *p =* 0.019) negatively affected QOL in this dimension.

## Discussion

This study measured the QOL of patients with CKD on hemodialysis and assessed the impact of various social, demographic, and clinical variables on the QOL of patients compared to controls.

The individuals included in the case group were predominantly males with incomplete middle school education (9.5 years of schooling) without a spouse. Years of schooling is a relevant variable, since it may serve as an indicator of the quality of the information provided by interviewees and the level of comprehension they had of the recommendations made to them by their healthcare providers, particularly in regards to care, life habits, and therapy.^5^

Our findings in the area of marital status did not agree with previous studies.^23^^,^^24^ Other authors^23^^,^^24^ found that most individuals on RRT had a spouse and lived with their families. For an individual with a chronic condition, the absence of a spouse may decrease the quality of care and deteriorate perceptions of QOL. In other words, spouses have been correlated with enhanced support to patients and improved QOL.^25^

The individuals in the case group were older than their counterparts in the control group. Their ages ranged from 21 to 81 years (mean age: 53.59 years) and were similar to the range seen in another study,^3^ in which case group members had a mean age of 54.71 years.

The two groups were economically different, with controls having higher levels of education, individual and household income. A study carried out in a hemodialysis center in Natal, in northeastern Brazil, found that most participants were pensioners.^6^ Another study^26^ conducted in the State of Rio de Janeiro reported household income levels from one to five minimum wages, with most individuals within the 1-2.5 minimum wage range. A study carried out in 2016 found that 42.9% of the patients were on leave from work on account of CKD and were being paid financial assistance by the National Institute of Social Security (INSS).^27^ With that in mind, the difference between group income levels may stem from the inability to work on the side of subjects with CKD arising from limitations and the time spent on hemodialysis.

More than half of the individuals in the case group had complications linked to CKD and hemodialysis, as described by other authors.^28^ The most frequent complications reported in our study were cardiovascular events, chronic anemia, calcium metabolism disorders, seizure, headache, nausea and vomiting, malaise, cramps, air embolism, and phlebitis, among others.

The WHOQOL-BREF scores of the individuals in the case group were significantly lower than the scores of the control group. Additionally, only two domains were statistically different between the case and control groups, namely the physical and psychological domains. Other authors have reported similar findings.^8^^,^^29^^,^^30^ The treatment of patients with CKD causes significant functional and physical impairment, manifested in issues such as sedentarism, poor socialization, loss of autonomy, and increased dependence on others, since they need help to perform a number of activities of daily living.

Individuals with CKD have trouble establishing and/or keeping employment on account of the time they spend on RRT, and suffer from physical impairment and symptoms such as weakness and malaise, which by their turn affect the performance of activities of daily living and produce psychological and emotional distress.^30^

The lower psychological domain scores attained by individuals in the case group sheds light on the emotional distress suffered by patients on dialysis. Symptoms arise and develop during therapy, to the point of limiting their ability to perform activities of daily living and compromising the emotional and personal aspects of their lives in the later stages of the disease, thus adversely affecting the perception patients have of their QOL.^31^

Other authors^32^^,^^20^ have reported high rates of psychological conditions such as depression, anxiety, and low self-esteem in patients on dialysis, which were associated with sexual dysfunction and other related complications.

Multiple linear regression analysis revealed that undergoing hemodialysis adversely affected the patients' level of satisfaction with their health status and the scores in the physical and psychological domains. Smoking negatively affected the perceptions of QOL of the two groups.

Other authors^33^^,^^34^ have described alterations and symptoms triggered by the disease and RRT and the restrictions and impairments to which patients are exposed in areas such as physical and functional health and overall wellbeing, all of which causally linked to decreased QOL.^35^

Patients undergoing expensive therapies experience physical and psychological distress and have significantly lower levels of satisfaction in their lives. Physical limitations, an uncertain future, and financial restrictions, to name a few, often favor the onset of depressive symptoms.^5^^,^^23^

According to Evaristo,^36^ smoking is a strong obstacle to optimal therapy management. A study^37^ designed to assess the impact of risk factors in chronic diseases evinced smoking as the risk factor with the single greatest impact on the QOL of patients.

Higher levels of education were linked to positive impacts on the physical and environmental domains, whereas having a spouse positively affected the social domain and higher income levels were associated with higher scores in the environmental domain.

Studies^5^^,^^21^ have shown that higher levels of education are linked to wider access to information and better economic status. Individuals with more years of schooling tend to have jobs that require less physical effort and are thus less affected by the disease at work. They also perform better at comprehending the information given to them by their caregivers and thus at complying with therapy.

The individuals on hemodialysis had lower QOL scores in the psychological domain. This finding may be explained by the repercussions of the disease, which go far beyond physical symptoms to generate emotional disorders such as anxiety, depression, reduced self-esteem, and other mental ailments.

The study^37^ found that most of the patients on hemodialysis had higher levels of anxiety due to the fact that they were connected to a dialyzer for several hours. Their self-image is severely impacted and negative feelings emerge with the implantation of a vascular access (arteriovenous fistula or catheter) that requires care and maintenance.^1^

In this study, the environmental domain was positively affected by individual income and years of schooling, while residing in the city of the hemodialysis center was a negative factor. This domain covers matters such as physical safety, availability of financial resources, opportunity to acquire new information, recreation and leisure, and healthcare availability.

Residing in the city where the study was carried out was also a relevant variable to negatively affect QOL. Study participants live in unsafe areas of the city and are less prone to leaving their homes by themselves, which makes them more susceptible to isolation and depression. They also face mobility issues connected with poor access to public transportation, which further decrease QOL.^38^

Multiple linear regression analysis of the scores of individuals on hemodialysis revealed that their perception of QOL was positively affected by the presence of a spouse and higher income levels. Conversely, undergoing hemodialysis at a public center and smoking were linked to perceptions of worse QOL.

Adversity appears to be better managed with the help of a spouse. Support from family and friends helps keep things balanced, encourages habit changes, and fosters behaviors that improve general health.^17^

The literature^39^ has singled out factors such as income and years of schooling as promoters of better health, since they may enable access to better care services and information, thus giving patients more autonomy and knowledge to engage in healthy behaviors and improve their wellbeing.^40^

Smoking has been described as a strong impediment to optimal therapy management.^41^ A study on the QOL of the elderly found that smoking was used as a means to escape loneliness, cheer up, sleep better, and even find relief from pain.^42^ With these factors in mind, one might understand why patients see smoking as a way to deal with CKD and its consequences. However, smoking has been associated with predisposition to other diseases, high levels of morbimortality, severe health problems, and decreased QOL.^43^

Undergoing hemodialysis at a public clinic had a negative impact on QOL. When referred to a higher complexity care center, patients understand that they are suffering from a severe disease and become more dissatisfied with their condition. Individuals referred to public care centers are usually poorer.^44^

Several studies have shown that the access and use of public health services mirror the realities of socioeconomic inequality in Brazil,^45^^,^^46^ since these services are primarily used by the poorer. Besides, patients experience the stress of constant compulsory visits to hospitals, in a process that deprives them of their independence and adversely affects their perceptions of QOL.^20^ These statements are suppositions, since the perceptions of individuals seen at public care centers versus the perceptions of their counterparts seen in private clinics have not been compared yet.

The following variables positively affected the physical domain: greater number of comorbidities; treatment at a public clinic; more years of schooling; older age; more persons in the household; and longer hemodialysis sessions. The number of comorbidities was negatively correlated with physical status, since the coexistence of various pathological conditions worsens the general health status while decreasing functional capacity and QOL.^37^ The study showed that years of schooling had a positive impact on QOL.^4^ In this study, individuals with CKD with more knowledge on the condition were more balanced and treatment compliant.

Unlike other studies, age was linked to higher physical domain scores. According to the literature,^22^ the impact of hemodialysis on pensioners is lower when compared to individuals of working age. Besides, the resilience acquired with years of treatment helps patients to accept their health status and working limitations.^47^

The number of persons in a household seemed to positively affect the physical domain, thus confirming that support from family and friends is relevant. The existence of a spouse/companion and/or residing with family may help with the care measures that have to be performed at home and activities of daily living.^25^ Older individuals usually rely more on the help of their families in the performance of activities of daily living, which contributes to the assignment of higher scores in the physical dimension of QOL.^30^

In the psychological domain, higher income had a negative effect on QOL. Although the literature cannot explain this finding, it has been suggested that individuals with higher levels of income have higher levels of education and are probably more aware of the implications of having CKD.^5^^,^^21^

This study found a correlation between longer hemodialysis sessions and higher scores in the physical, social, and environmental domains. A possible explanation for the correlation with the physical domain is that individuals on longer hemodialysis sessions have lower levels of nitrogenous wastes and experience more significant decreases in the symptoms of uremia, which together improve their physical condition.^48^ According to the study,^49^ longer hemodialysis sessions allow for less intense ultrafiltration and decreased muscle cramps.

In the social and environmental domains, longer hemodialysis sessions may also mean that patients have more time to engage with their peers with CKD. The time spent on hemodialysis is thus used to talk to other patients and healthcare workers about their emotions and fears.^50^ This may also affect other variables such as loneliness, having fewer ties of affection, and having more comorbidities such as diabetes mellitus, heart failure, and other cardiovascular diseases. 

Interestingly, residing in the same city of the hemodialysis center led to lower scores in the physical, psychological, and environmental domains. In the urban setting where the study was carried out - a mid-size city - the quantity and intensity of ties with other individuals is lower (and often inexistent), differently from what happens in smaller towns, in which interpersonal relationships occur in greater numbers and more intensely. In large cities factors such as urban violence, lack of security, traffic, and other stressors promote social isolation and the onset of mood disorders.^33^

In such circumstances, individuals are less prone to walking on their own and become more susceptible to isolation and mobility issues, which may impact all dimensions of QOL in persons with CKD.

Defining QOL based on only one generic instrument is not enough to measure the actual magnitude of the impact of the disease in the lives of patients. Studies with a larger and more representative number of individuals are required to fill the gaps exposed in this study. The authors further recommend that these points be addressed in future studies in order to more profoundly explain the impact on QOL variables and the possible cause-and-effect mechanisms.

## Conclusion

The WHOQOL-BREF scores of patients with CKD on hemodialysis were lower than the scores observed in the control group. Only the scores in the physical and psychological domains were statistically different between the case and control groups.

The variables that more significantly affected the QOL of individuals with CKD on hemodialysis were having a spouse, the number of comorbidities, undergoing hemodialysis at a public clinic, more years of schooling, older age, living with more persons in the household, and longer hemodialysis sessions.
